# The oncolytic peptide LTX-315 induces cell death and DAMP release by mitochondria distortion in human melanoma cells

**DOI:** 10.18632/oncotarget.5308

**Published:** 2015-10-13

**Authors:** Liv-Marie Eike, Nannan Yang, Øystein Rekdal, Baldur Sveinbjørnsson

**Affiliations:** ^1^ Department of Molecular Inflammation Research, Department of Medical Biology, Faculty of Health Sciences, University of Tromsø, Tromsø, Norway; ^2^ Department of Community Medicine, Faculty of Health University of Tromsø, Tromsø, Norway; ^3^ Lytix Biopharma, Oslo, Norway

**Keywords:** oncolytic peptide, DAMPs, melanoma

## Abstract

Host defense peptides (HDPs) are naturally occurring molecules found in most species, in which they play a significant role in the first line defense against intruding pathogens, and several HDPs have been shown to possess anticancer activity. Structure-activity relationship studies on the HDP bovine lactoferricin revealed a *de novo* design of a nonamer peptide LTX-315, with oncolytic properties.

In the present study, we investigated the oncolytic activity of LTX-315 in human melanoma cells (A375). LTX-315 induced a rapid plasma membrane disruption and cell death within 2 hours. At a low concentration, fluorescence-labeled LTX-315 was internalized and accumulated in cytoplasmic vacuoles in close proximity to the mitochondria. The mitochondrial membrane potential was shown to depolarize as a consequence of LTX-315 treatment and at ultrastructural level, the mitochondria morphology was significantly altered. Release of danger signals (DAMPs) such as ATP, Cytochrome C and HMGB1 into the cell supernatant of cultured cells was evident minutes after peptide treatment.

The oncolytic effect of LTX-315 involving perturbation of both the cell membrane and the mitochondria with subsequent release of DAMPs may highlight the ability of LTX-315 to induce complete regression and long-term protective immune responses as previously reported in experimental animal models.

## INTRODUCTION

**Figure 1:**
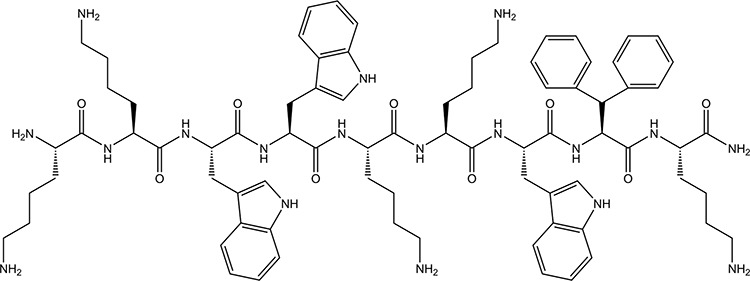
Chemical structure of LTX-315 (K-K-W-W-K-K-W-Dip-K-NH2) generated using ChemDraw 11 Dip is the aromatic non-coded amino acid β-diphenylalanine.

Host defense peptides (HDPs) represent a diverse group of peptides found in virtually all species of life as a part of the innate immune system [1, 2]. In addition to the bactericidal effects, many HDPs have been shown to have antitumor [3] and antiviral [4] activity, and contribute to both innate and adaptive immunity [5, 6]. The host defense peptide lactoferricin (LfcinB) is a cyclic 25-amino acid peptide widely investigated for both its antimicrobial properties, as well as for antitumor effect, *in vitro* and *in vivo* [7–9]*.* Based on extensive structure-activity studies performed on LfcinB, we have identified several structural parameters critical to its anti-tumor activity and selectivity [10–12]. With an optimization of these parameters, a new group of shorter and more potent anticancer peptides has been designed. One of these, LTX-302, was reported to rapidly induce necrosis in murine cancer cells [13]. Interestingly, LTX-302 treatment also induced a complete regression and subsequent protection against re-challenge in an experimental animal model by inducing an adaptive immune response [13]. We have recently reported anticancer effects of the nonamer LTX-315 (Figure 1) [14, 15], which is considerably shorter than the model peptide LfcinB (25aa). LTX-315 has the ability to adopt a α-helical secondary structure and contains five cationic Lys residues, three Trp residues, the bulky non-coded residue β-diphenylalanine (Dip) and an amidated C-terminal. This peptide has been shown to rapidly induce necrosis and anticancer immune responses after intratumoral treatment in an experimental murine melanoma model [14, 15]. Given the strong immunomodulatory effect of LTX-315 observed *in vivo*, we wanted to investigate the mechanisms of action of LTX-315 in human melanoma cells *in vitro*.

## RESULTS

### Cytotoxic effect of LTX-315 on melanoma cells

**Figure 2:**
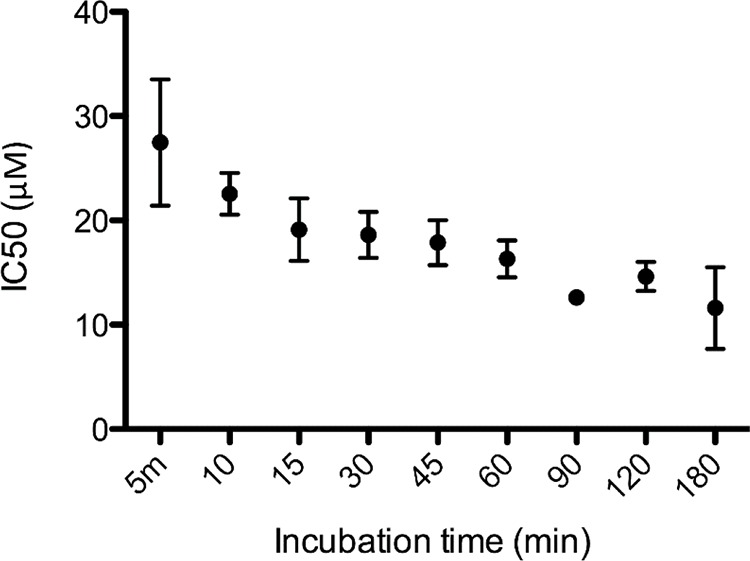
LTX-315 causes rapid cell death in human melanoma cells *In vitro* cell killing kinetics of LTX-315 (IC_50_) against human melanoma cells after designated time points.

The cytotoxic activity of LTX-315 on A375 melanoma cells was investigated at different time points. The IC_50_ values determined at each time point show that LTX-315 killed the cancer cells within minutes. (Figure 2)

The IC_50_ value for LTX-315 was 30 μM after only five minutes of exposure and lowered to 17 μM after 60 minutes.

### LTX-315 treatment causes rapid cell lysis

**Figure 3:**
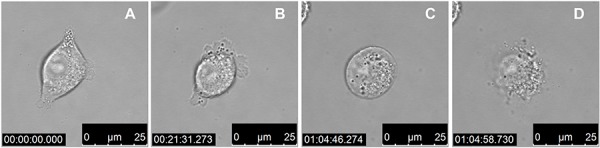
LTX-315 kills human melanoma cells in a lytic mode of action Bright field confocal images of A375 cells treated with 17 μM LTX-315. **A.** 1 min after added peptide, **B.** 22 min after added peptide, **C.** 65 min after added peptide, **D.** 65 min after added peptide (12.5s after **c**)

We next wanted to assess the cell morphology of A375 melanoma cells after being treated with LTX-315. Cells were treated in a time dependent manner with LTX-315 (17 μM) and investigated by bright field confocal microscopy. Treated cells displayed a rapid change from a normal epithelial morphology to a total collapse of the cells with an extrusion of cytoplasmic content, which was proceeded by a rounding up of the cell (Figure 3). These changes occurred in the majority of cells within 15–60 minutes of treatment with LTX-315. A time-lapse movie showing the morphological changes in treated cells is enclosed in the supplementary section.

### LTX-315 rapidly induced loss of plasma membrane integrity

**Figure 4:**
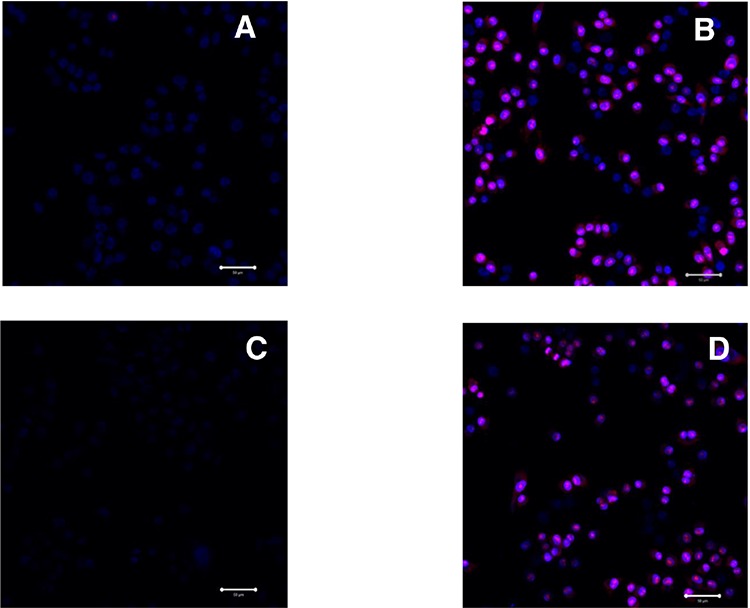
LTX-315 decrease plasma membrane integrity independent of temperature; A375 cells treated with 35 μM LTX-315 and labelled with PI (red) and nuclear dye Hoechst 33342 (blue) was investigated using confocal microscopy **A.** untreated cells at 37°C, **B.** treated cells at 37°C, **C.**) untreated cells at 4°C, **D.** treated cells at 4°C.

To further investigate the membranolytic activity of LTX-315, treated cells were labeled with the DNA binding fluorescent probe PI. This dye is commonly used to distinguish between live and necrotic cells in various assays as it only enters cells with a compromised plasma membrane. To elucidate if the initial membranolytic effect was dependent on internalization of the peptide we conducted the following experiments at 4°C and 37°C respectively. Live cell imaging with confocal microscopy demonstrated that treatment LTX-315 for 5 minutes was sufficient to induce loss of plasma integrity in a majority of cells at both 4°C and 37°C (Figure 4A–4D). Hence, the membranolytic activity by LTX-315 is not dependent on endocytosis or other types of active transportation mechanism.

### LTX-315 internalizes and targets the mitochondria

**Figure 5:**
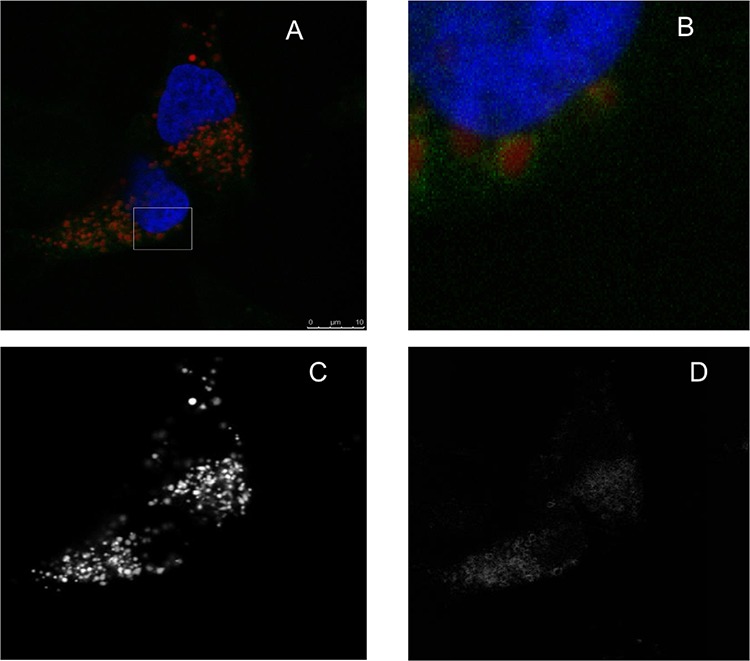
LTX-315 internalizes and accumulates close to the mitochondria A375 cells were transfected with fluorescent organelle markers. The mitochondria were labeled using the pDsRed2-Mito, and the nucleus was labeled using the GFP-Histon2B plasmid, prior to treatment with fluorescence labeled LTX-315 peptide (Pacific Blue). Transfected cells were treated 30 minutes with 1,5 μM fluorescence-labeled LTX-315 (green), and with labeled mitochondria (red) and nucleus (blue pseudo color). The peptide was internalized and detected in close proximity to the mitochondria. **A.** overlay channels, **B.** close up, **C.** mitochondria. **D.** LTX-315.

**Figure 6:**
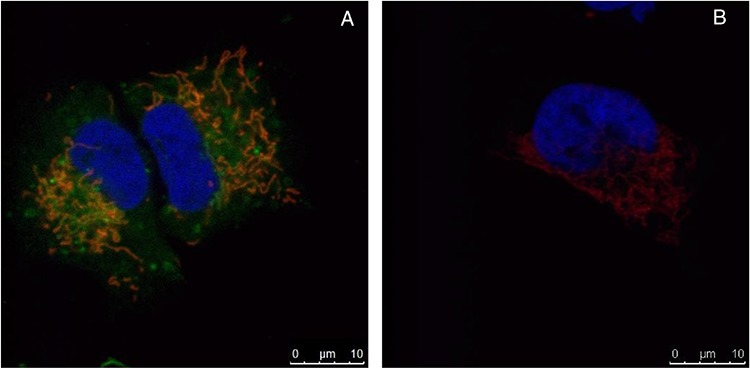
Internalization occurs only in labeled LTX-315 and not in the labeled mock peptide LTX-328 A375 cells treated with 35 μM LTX-315 **A.** or LTX-328 **B.** peptide for 60 min. Fluorescence-labeled peptide (green), and with labeled mitochondria (red) and nucleus (blue pseudo color). LTX-315 was detected in the cytoplasm, while LTX-328 was not internalized.

**Figure 7:**
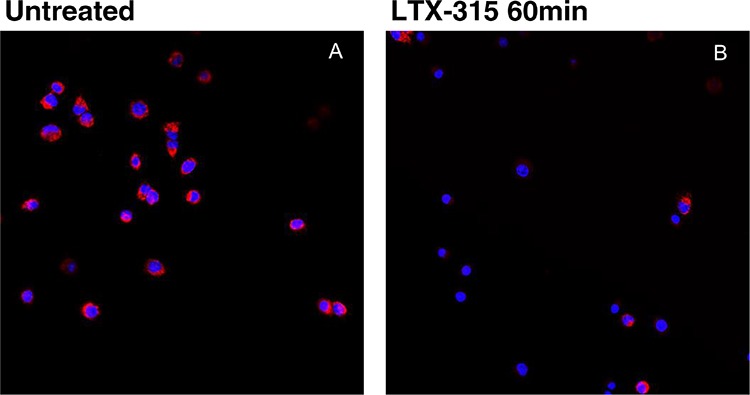
LTX-315 causes decreased mitochondrial membrane potential A375 cells treated with 17 μM LTX-315 for 60min were subsequently labelled with Mitotracker. A lower signal was detected in the treated cells **B.** as compared with untreated cells **A.** (Z-stack, maximum intensity projection)

To investigate if LTX-315 is internalized and has any intracellular targets, A375 cells were incubated with Pacific Blue-labeled LTX-315. The labeled LTX-315 rapidly penetrated the plasma membrane and showed an accumulation close to the mitochondria after 30 minutes of incubation without being detected in the cell nucleus (Figure 5). The labeled non-lytic mock-sequence peptide LTX-328 did not demonstrate any internalization at any concentration or incubation time tested (Figure 6B). LTX-315 treated A375 cells also showed a decreased signal from the mitochondrial membrane potential sensitive dye Mitotracker. (Figure 7), indicating that LTX-315 interacts with the mitochondria membrane.

### LTX-315 induces ultra-structural changes in cells

**Figure 8:**
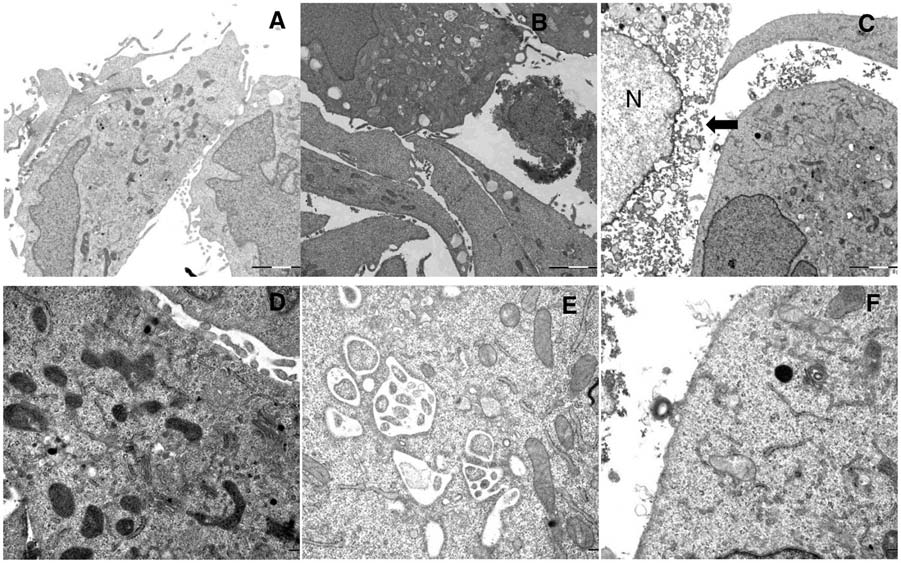
Ultrastructural changes in cells treated with LTX-315 TEM images of A375 cells treated with LTX-315 for 60 minutes compared to control cells. **A. D**: untreated control cells, **B. E.**: cells treated with 3,5 μM, **C., F.** cells treated with 17 μM, arrowhead indicate cell with disintegrated plasma membrane, N indicate nucleus. Magnification 10 000X **A-C**, 30 000 **D-F**.

We further evaluated the ultra-structural changes in A375 cells after LTX-315 treatment by performing transmission electron microscopy (TEM). A significant number of the cells treated with a low concentration of the LTX-315 peptide (3,5 μM) for 60 minutes showed Vacuolization, as well as altering of the mitochondrial morphology (Figure 8B). The mitochondria appeared to be less electron-dense, also exhibiting some degree of reorganization, with the cristae lying further apart or not visible at all. This in contrast to electron dense, normal appearing mitochondria of the untreated cells. (Figure 8A and 8D) The number of necrotic cells in these samples was less than 5%. Moreover, perinuclear vacuoles were also observed at low concentration of LTX-315, with a content consisting of cytosolic material such as ribosomes and other organelles (Figure 8E). Another common finding in these samples were peripherally placed vacuoles, which were lined with a single membrane layer containing a homogenous material (Figure 8B). When cells were treated with a higher concentration (17 μM) for 60 min, approximately 40% of them displayed a necrotic morphology with a loss of plasma membrane integrity (Figure 8C). The cells that were still intact displayed a great heterogeneity, from a normal appearance with microvilli to a round appearance, with mitochondria clearly affected. At this high concentration, only 4% of the cells investigated displayed vacuolization, and chromatin condensation was not visible in the cells at any peptide concentration tested. These results demonstrate that LTX-315 kills the tumor cells with a lytic mode of action, while lower concentrations cause the cells to undergo ultrastructure changes, such as vacuolization and an altered mitochondrial morphology. No significant morphological changes suggestive of apoptotic cell death were observed.

### LTX-315 treatment leads to extracellular ATP release

**Figure 9:**
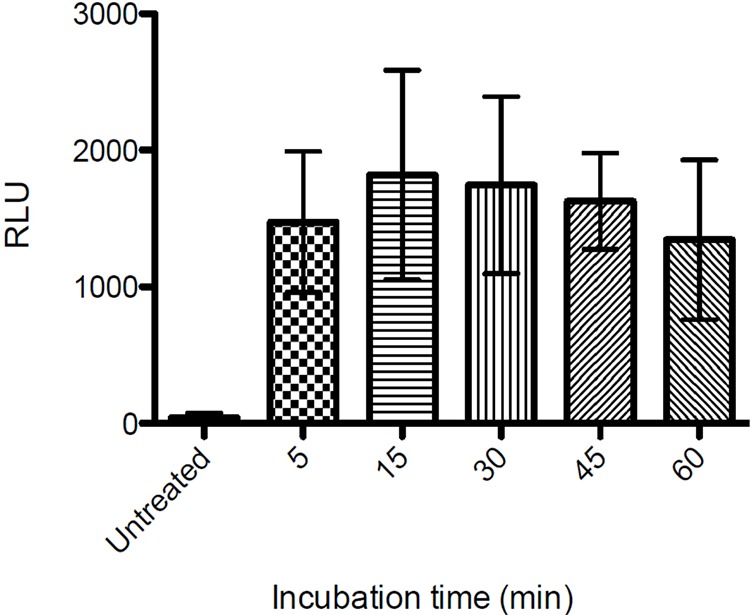
Extracellular ATP levels following LTX-315 treatment A375 cells were treated with LTX-315 (35 μM) for different time point or maintained under controlled conditions, and supernatant analyzed for the quantification of ATP secretion by luciferase bioluminescence. Quantitative data (mean +- S.D.) for one representative experiment are reported.

Danger-associated molecular pattern molecules (DAMPs) are molecules that are released from intracellular sources during cellular damage. DAMPs can initiate and perpetuate an immune response through binding to Pattern Recognition Receptors (PRRs) on Antigen Presenting Cells (APCs). Among commonly known DAMPs are ATP, HMGB1, Calreticulin, Cytochrome C, mitochondrial DNA and Reactive oxygen species (ROS) [18–20]. We investigated release of several DAMPs including the level of ATP released into the supernatant from cells treated with LTX-315. 35 μM The supernatant from treated and non-treated cells was diluted at 1:10 and analyzed using luciferase detection assay. As shown in Figure 9, ATP was detected in the supernatant as early as after 5 minutes of treatment with LTX-315.

### LTX-315 treatment induces cytochrome-C release in supernatant

**Figure 10:**
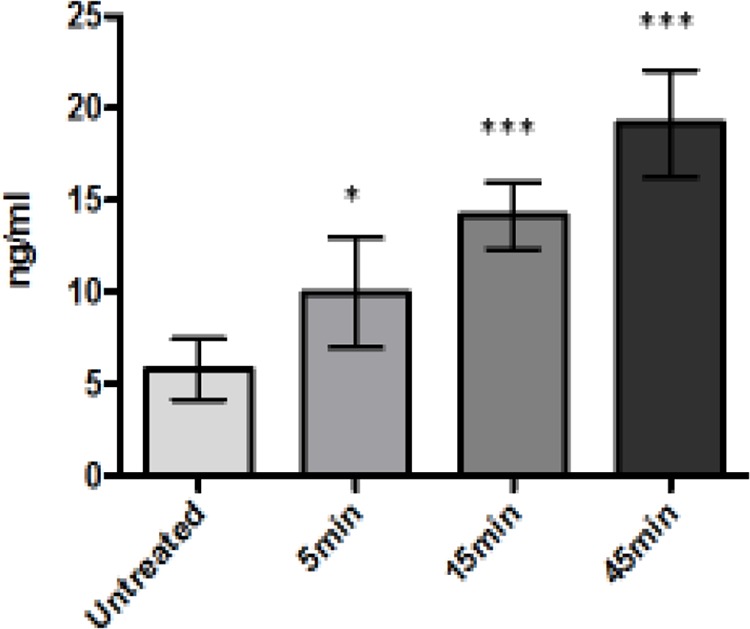
A375 melanoma cells treated with LTX-315 35 μM release cytochrome-C into the cell supernatant Cytochrome-C was detected by Elisa assay.

To assess whether LTX-315-treated cells released cytochrome-C into the medium, A375 cells were treated with LTX-315 (35 μM) at different time points (5, 15, 45 min). The supernatant was subsequently analyzed for cytochrome-C levels using an ELISA assay. Cell cultures treated with 35 μM had a 3-fold increase of cytochrome-C in the supernatant compared to untreated control cells. The increase in cytochrome-C was detected already at five minutes of exposure with LTX-315. (Figure 10).

### LTX-315 treatment leads to extracellular HMGB1 release

When the nuclear protein HMBG1 is released into the extracellular fluid, it functions as a DAMP, and can bind to both the PRR TLRs and to the RAGE receptors; whose activation leads to a number of inflammatory responses such as activation and maturation of antigen presenting cells (APCs). In order to assess the release of HMGB1 from LTX-315-treated cells, we measured the translocation of HMGB1 from the nuclear compartment into the culture supernatant [18]

**Figure 11:**
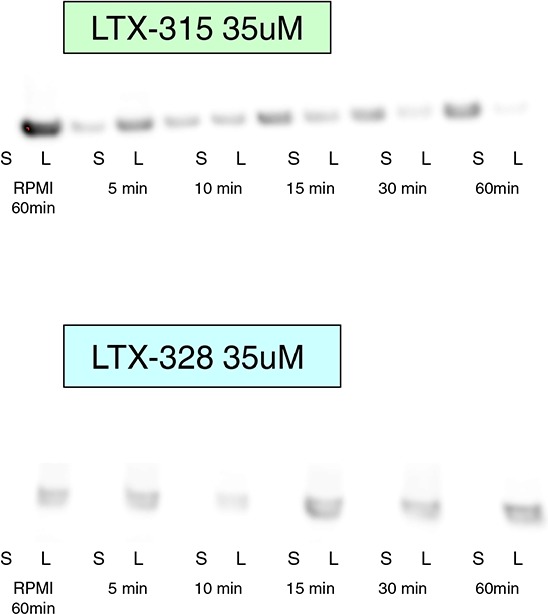
HMGB1 is released in the supernatant after LTX-315 treatment A375 human melanoma cells were treated with 35 μM LTX-315 (top) or LTX-328 (bottom), and cell lysate **(L)** and supernatant **(S)** were analyzed with western blot. HMGB1 from LTX-315-treated cells demonstrated a gradual translocation from the cell lysate to the cell supernatant. Control cells were treated with media alone, and showed no translocation after 60 minutes.

Both cell lysate and the cell supernatant of LTX-315- and LTX-328-treated A375 melanoma cells were analyzed using a western blot. Cells were treated with 35 μM of either LTX-315 or LTX-328, and a gradual increase in translocation of HMGB1 from the cell lysate to the supernatant was detected in the LTX-315-treated melanoma cells, but not in the supernatants of cells treated with the mock sequence peptide LTX-328 or a serum-free medium only (Figure 11).

### LTX-315 treatment causes the production of Reactive Oxygen Species (ROS) in A375 melanoma cells

**Figure 12:**
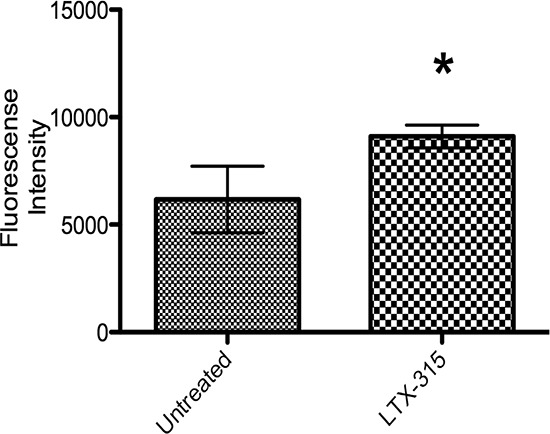
ROS generation in LTX-315 induced cell death A375 cells were treated with LTX-315 17 μM at different concentrations for 30 minutes. After peptide treatment, carboxy-H2DCFDA was added to the samples and fluorescence was analyzed with a fluorescence plate reader. The experiment was conducted in duplicate, with bars representing mean fluorescence +- S.D.

Reactive Oxygen Species (ROS) have been reported to play important roles in immunogenic cell death [19, 20], and one important source of ROS can be dysfunctional mitochondria [21, 22]. It has also been implied to decrease the immunogenicity of released HMGB1 by oxidation [23]. The ROS generation following LTX-315 treatment was measured by CH2DCFDA fluorometric assay. An increase in ROS production was generated after 15 minutes of incubation with LTX-315. (Figure 12).

## DISCUSSION

Recent advances in immunotherapy treatment of metastatic melanoma has gained much attention lately [24]. Especially the overwhelming and lasting clinical response in some patients treated with immune checkpoint blockade, has been the source of growing optimism and interest in the field of cancer immunotherapy [25, 26]. However, the response rates in patients with metastatic melanoma treated with these new drugs are typically below 40% and a significant number of patients experience serious adverse effects. [27, 28] More insight into beneficial combinations of existing treatments are called for, as well as biomarkers for successful patient selections. [29–31] There is also interest in new approaches within cancer immunotherapy. Host defense peptides (HDPs) are a part of the innate immune system [1]. There is a wide range of structural diversity between different HDPs, but common features are cationicity, amphipatic conformation, and an ability to interact with cellular membranes [1, 32]. Some HDPs and *de novo* designed peptides have been shown to exhibit anticancer activities in a number of experimental studies [3, 33-35], and the most commonly described mode of action is a direct membranolytic effect [36]. Several studies of cancer cells from different cancers demonstrate that the plasma membrane of malignantly transformed cells are different from their normal counterparts with regard to overall electrical charge, membrane fluidity and cell surface area. These changes makes cancer cells more vulnerable and allows for a more selective killing of cancer cells as compared to normal cells. Cancer cells are known to be more negatively charged than the membranes of their normal counterpart cells, due to a higher amount of anionic components in the plasma membrane such as phosphatidylserine [37–39], proteoglycans with heparin sulphate [40–42] and sialic acid on glycoproteins (e.g. mucins) [43–45]. These changes may cause cancer cells to be vulnerable and allows for a more selective killing of cancer cells by cationic peptides as compared to normal cells. The naturally occurring peptide LfcinB has served as a model peptide for a new group of shorter oncolytic peptides [13].

Intratumoral treatment with one of these peptides, LTX-315, was shown to induce tumor necrosis and long-lasting regression in in the majority (80%) of animals in a murine B16 melanoma model [15]. Histological investigations of treated tumors revealed extensive hemorrhagic necrosis and infiltration of CD3^+^ T cells. Moreover, mRNA levels of inflammatory cytokines such as IL1β, IL6 and IL18 were found to be augmented in the tumor tissue after LTX-315 treatment. The treatment did also prevent lung metastasis in mice re-challenged with B16F1 cells intravenously [15]. Tumor protective immune responses developed by another short oncolytic peptide involved both CD4+ and CD8+ T cells (13). Taken together these findings demonstrates that oncolytic peptides have an ability to stimulate strong T-cell dependent protective cancer specific responses. In this article, we investigate the mode of action of the oncolytic peptide, LTX-315 in more detail *in vitro*. *In vitro* studies demonstrated that LTX-315-induced cell death in A375 melanoma cells takes place within minutes after treatment. (Figure 2). This stands in contrast to conventional cytostatic drugs, which target the cell's DNA or mitotic apparatus, killing dividing cells in a matter of several hours or days [46, 47]. In general, HDPs are known to kill cancer cells by either plasma membrane attack via pore formation or through membrane destabilization, as well as by the induction of programmed cell death, independent of proliferative status or drug-resistant phenotype [3]. The ability to induce loss of plasma membrane integrity by penetration or destabilization is a common feature within the diverse classes of HDP [2, 3, 34, 48], and LTX-315 proved to share this feature as demonstrated with PI studies performed with confocal microscopy. Cells treated with 35 μM LTX-315 were PI positive after 5 min, demonstrating a rapid effect against the plasma membrane (Figure 4). To determine if the process of plasma membrane disruption was temperature dependent, parallel experiments at both 4°C and 37°C were performed. We observed that LTX-315 was able to disintegrate the plasma membrane at low temperature, pointing toward a direct interaction with the plasma membrane (Figure 4). Confocal and electron microscopy imaging of LTX-315-treated cells supported a direct membrane effect since a majority of the cells treated with a high concentration of LTX-315 (17 μM) was killed by a membranolytic mode of action (Figures 3, 4). The parental peptide LfcinB has been proven to internalize and cause cell death by targeting the mitochondria, resulting in both caspase-dependent and caspase independent cell death [8, 9, 49]. These findings, together with the knowledge that the mitochondria are the most negatively charged organelle in the cytoplasm and would therefore likely attract any internalized cationic-charged molecule, led us to investigate whether LTX-315 was internalized and targeted to the mitochondria. Indeed, by labeling with the fluorescent molecule Pacific Blue, LTX-315 was shown to be internalized and distributed in the cytoplasm. At low concentrations, accumulation of the peptide in close proximity to the mitochondria was evident, whereas at higher concentrations the peptide was more even distributed within the cytoplasm and accumulated in circular structures both in association to the mitochondria but also closer to the cell membrane (Figure 6A). The fluorescence labeled mock sequence peptide LTX-328 was not detected intracellularly, proving that internalization is specific for LTX-315 and not caused by the fluorescent tag. A confocal imaging of the melanoma cells labeled with the membrane potential-dependent mitochondrial stain Mitotracker CMXh2ROS showed a loss of mitochondrial signal 60 minutes after peptide treatment (Figure 7), indicating that the peptide targets the mitochondria membrane and causes a loss of mitochondrial membrane potential. Cell morphology studies (TEM) showed significant alternations in the mitochondria morphology after LTX-315 treatment. Treated cells demonstrated less electron-dense mitochondria with a distortion of the cristae organization, as well as vacuolization within the mitochondria as compared to mitochondria of untreated cells (Figure 8 A–8C). Furthermore, vacuolization within the cytoplasm was evident in approximately 20% of cells treated with 3,5 μM of LTX-315. Hence, these findings reveal that the de novo generated LTX-315 peptide is internalized and disintegrate the mitochondria.

When the mitochondria are dysfunctional, free oxygen radicals (ROS) may be formed [21]. An increased ROS formation was detected within a few minutes after LTX-315 treatment (Figure 12). However, the fluorometric assay used is not specific for mitochondrial ROS and may measure other intracellular sources of ROS such as ER and peroxisomes [50]. Independent of intracellular source an increase in ROS may lead to cell death [51, 52] through oxidation of lipids and proteins, as well as DNA damage and mitochondrial dysfunction [53]. ROS levels have also been linked to cell death by autophagy, a self-catabolic process in which the cell engulfs and destroys its own organelles via lysosomal proteinase-regulated elimination [53].

DAMPs (Death Associate Molecular Pattern) are naturally expressed molecules that signal tissue damage to the host. These molecules may be secreted by activated macrophages, natural killer (NK) cells or mature dendritic cells (DC) in response to inflammatory stimuli such as infection or tissue injury, or are released into the extracellular compartment by distressed or necrotic cells [54]. DAMPs exert strong stimulatory effect on immune cells. There are a high number of recognized and suggested DAMPs such as High Motility Group Box 1 proteins (HMGB1), Adenosine Triphosphate (ATP) [55–59] and mitochondrial proteins such as Cytochrome-C and also ROS generated by damaged or dysfunctional mitochondria [60, 61]. DAMPs can bind to Pattern Recognition Receptors (PRRs) on the cell surface or in the cytosol, thus leading to pro-inflammatory cytokine production and the activation of antigen presenting cells [62]. The mitochondria are important mediators of inflammation [60, 61, 63, 64] and a source of DAMPs [22, 63, 65, 66]. Since LTX-315 treatment affects the mitochondria, its ability to induce release of certain mitochondrial derived DAMPs such as ATP and Cytochrome-C was investigated.

Increased levels of Cytochrome-C were detected in the supernatant of LTX-315 treated cell cultures (Figure 10). Cytochrome-C is a mitochondrial protein released from the intermembrane space and into the cytosol when the outer mitochondrial membrane is perturbed. By binding to the apoptotic protease activating factor-1 (Apaf-1) it is also a part of the apoptotic cascade that eventually leads to cell death by apoptosis. However, when cytochrome-C is present in the extracellular space, it has been reported to act as a pro-inflammatory mediator, thus activating NF-*k*B and inducing cytokine and chemokine production [67]. We also detected increased levels of ATP in the supernatant of LTX-315 treated cells (Figure 9). When ATP is released extracellularly it functions as a DAMP by activating the purinergic P2RX7 receptors on dendritic cells (DC) [55]. This receptor not only functions as a pore that opens for small cationic and later larger molecules after binding to ATP, its activation also causes the processing and release of the pro-inflammatory cytokine IL-1β [68, 69].

High-Mobility-Group-Box-1 (HMGB1) is a non-histone, chromatin-binding nuclear protein. Once passively released from necrotic cells, HMGB1 is able to trigger the functional maturation of dendritic cells, cytokine stimulation and chemotaxis among several immunopotentiating effects. HMGB1 is normally present in the cell nucleus and would be expected in a cell lysate of untreated cells, and not in the culture supernatant. [22, 56, 70, 71]. Western blot analysis of treated A375 cultures showed a translocation of HMGB1 from the cellular compartment to the supernatant (Figure 11), thus revealing that LTX-315 treatment can cause release of non-mitochondrial DAMPs.

Taken together, our data demonstrate that LTX-315 induces lytic cell death in cancer cells, not only by directly attacking the plasma membrane, but also as a result of an injury to vital intracellular organelles, particularly mitochondria, after the internalization of the peptide at concentrations too low to cause an immediate loss of plasma membrane integrity. We demonstrate that the peptide treatment causes the release of several DAMPs such as cytochrome-C, ATP, HMGB1. The DAMPs may affect the cellular integrity of the damaged cells in several ways, but are also associated with hallmarks of so-called immunogenic cell death [72–74]. The excessive release of DAMPs may help explain the strong effect of LTX-315 obtained in experimental animal tumor models, which have not only demonstrated a primary tumor regression and lymphocyte infiltration, but also protective and systemic immune response upon re-challenge with the same tumor [14, 15]. The overall strong therapeutic effect may be due to the release of tumor-specific antigens released into the extracellular compartment, together with potent immune stimulatory molecules (DAMPS) such as ATP, cytochrome-C and HMGB1. In turn, these will lead to a maturation and activation of DCs and other accessory cells of the adaptive immune system. Our data unveil the oncolytic effect of LTX-315, involving perturbation of both the cell membrane and the mitochondria, with subsequent release of DAMPs, which highlight the ability of LTX-315 to induce complete regression and long-term protective immune responses. LTX-315 has a potential as a novel immunotherapeutic agent and is currently tested in clinical phase I as a first in class oncolytic peptide immunotherapy.

## MATERIALS AND METHODS

### Reagents

LTX-315 (K-K-W-W-K-K-W-Dip-K-NH2) and LTX-328 (K-A-Q-Dip-Q-K-Q-A-W-NH2) were made on request by Bachem AG (Bubendorf, Switzerland) and Innovagen (Lund, Sweden), respectively. Dip is the aromatic non-coded amino acid β-diphenylalanine. LTX-315 Pacific Blue and LTX-328 Pacific Blue were purchased on request from Innovagen (Lund, Sweden) and Norut (Tromsø, Norway), respectively.

### Cell culture

The A375 cell line A375 (ECACC, 88113005) is a human malignant melanoma derived from patient material, and was purchased from Public Health England (PHE Culture Collections, Porton Down, Salisbury, UK). Cells were maintained as monolayer cultures in high glucose 4.5% DMEM supplemented with 10% FBS and 1% L-glutamine, but not as antibiotics (complete medium). The cell line was grown in a humidified 5% CO_2_ atmosphere at 37°C, and was regularly tested for the presence of mycoplasma with MycoAlert (Lonza).

### *In vitro* cytotoxicity

The cytotoxic effect of LTX-315 was investigated using the colorimetric MTT viability assay [8, 16, 17]. The A375 cells were seeded at a concentration of 1 × 10^5^ cells/ml in a volume of 0.1 ml in 96-well plates, and allowed to adhere in a complete growth medium overnight. The media was then removed and the cells were washed twice in serum-free, RPMI-1654 medium, before adding LTX-315 dissolved in serum-free RPMI at concentrations ranging from 2-180 μg/ml, and incubated for 5–180 minutes. Cells added serum-free RPMI were used as negative control cells, while cells treated with 1% Triton X-100 in serum-free medium were used as a positive control. The results were calculated using the mean of three experiments, each with triplicate wells.

### Confocal microscopy

*Live cell imaging with unlabeled cells -* A375 cells were seeded at 10,000 cells/well in a complete medium in Nunc Lab-Tec 8-wells chambered covered glass (Sigma) pre-coated with 25 μg/ml human fibronectin (Sigma) that were allowed to adhere overnight. Cells were washed twice with a serum-free RPMI, treated with peptide dissolved in RPMI and investigated using Bright Field on a Leica TCS SP5 confocal microscope, with a 63X/1.2W objective. The microscope was equipped with an incubation chamber with CO_2_ and temperature control.

*Live cells, mitotracker* - Cells were seeded as for live cell imaging, and treated with Mitotracker CMH2XROS (Invitrogen) at 100 nM for 15 minutes prior to peptide treatment. Cells were treated with 17 μM LTX-315, with negative control serum-free RPMI only. After 60 min of incubation, cells were analyzed using a Zeiss microscope equipped with an incubation chamber with CO_2_ and temperature control.

experiments were subsequently conducted at least twice with similar results.

*Live cell- propidium iodide* – A375 cells were seeded at 10,000 cells/well in a complete medium in Nunc Lab-Tec 8-wells chambered covered glass (Sigma) and allowed to adhere 48 hours. Cells were washed twice with a serum-free RPMI, treated with 35 μM LTX-315, propidium iodide (PI) (sigma) and Hoechst33342 dissolved in RPMI. Incubation time was 5 minutes and was performed in regular incubation condition or on ice. The media was subsequently replaced with serum free RPMI before immediate analyzation using a Zeiss confocal microscope equipped with an incubation chamber with CO_2_ and temperature control.

*Fixed cells, fluorescence-labeled peptide -* Subconfluent A375 cells were seeded at 8,000 cells/well as above, and transfected the second day using the Lipofectamine LTX with Plus transfection reagents (Invitrogen) following the manufacturer's protocol. The mitochondria were labeled using the pDsRed2-Mito, and the nucleus was labeled using the GFP-Histon2B plasmid (a kind gift from the Imaging Platform, University of Tromsø). A day after transfection, cells were washed twice with serum-free RPMI, and treated at different concentration and incubation periods with LTX-315 Pacific Blue or LTX-328 Pacific Blue. LTX-315 PB exhibited a similar cytotoxic profile as the unlabeled LTX-315 as determined by MTT assay. Control cells were treated with unlabeled LTX-315 and also with serumfree RPMI only. After incubation, cells were fixed with 4% paraformaldehyde in PBS, and the wells were covered with Prolong Gold antifade (Invitrogen). Cells were further analyzed by use of a Leica TCS SP5 confocal microscope, with a 693, 1.2 W objective. Pacific Blue, GFP and Ds Red were excited using UV, with 488 and 561 lasers, and fluorescence channels were sequentially detected using the following band passes: UV: 420–480 nm (with attenuation), 488: 501–550 nm and 561: 576–676 nm.

### TEM electron microscopy

A375 cells were seeded at 1 × 10^5^ cells per well in 6-well plates and allowed to grow for three days to optimize membrane structures in the culture, and the medium was changed on the second day. Cells were washed twice in serum-free RPMI before being treated with LTX-315 dissolved in serum-free RPMI at 3.5 and 17 μM, with serum-free RPMI as a negative control. Cells were then washed with PBS before fixation for 24 hours at 4°C with 4% formal aldehyde and 1% gluteralaldehyde in a Hepes buffer at pH 7.8. Dehydration and post-fixation protocols included incubation in a 5% buffered tannic acid and incubation in 1% osmium-reduced ferrocyanide. Ultrathin sections were prepared, and uranyl acetate (5%) and Reynolds's lead citrate were used for staining and contrasting. Samples were examined on a JEOL JEM-1010 transmission electron microscope, and images were taken with an Olympus Morada side-mounted TEM CCD camera (Olympus soft imaging solutions, GmbH, Germany).

### Fluorescence measurement of reactive oxygen species (ROS)

A DCFDA cellular reactive oxygen species detection assay kit was purchased from Abcam^®^ (UK), and A375 cells seeded in a 96-well Costar black clear bottom plate with 20,000 cells per well incubated in 37°C 16 hours prior to DCFDA assay. Cells were washed with a 100 μL/well of pre-warmed PBS one time, and incubated with 20 μM of DCFDA in a buffer solution supplied with the kit at 37°C in a cell culture incubator for 45 min, and then washed again with a buffer solution of 100 μL/well. The cells were subsequently incubated with a 100 μL/well LTX-315 peptide dissolved in HBSS buffer solution at concentrations of 17 μM for 30 min, and untreated cells were used as a negative control. The fluorescence intensity was determined at an excitation wavelength of 485 nm and an emission wavelength of 530 nm on a FLUOstar Galaxy plate reader.

### Release of high mobility-group box-1 (HMGB1)

A375 cells were seeded with 3 × 10 ^5^cells/well in 6-well plates in complete medium, and allowed to adhere overnight. Cells were treated with LTX-315 or LTX-328 at 35 μM, and incubated at 37°C and 5% CO_2_ for different time points (5, 10, 15, 30, 60 min), and negative controls were serum-free RPMI-1650. Supernatants (S) were collected and centrifuged at 1,400 g for five minutes, and cell lysates (L) were harvested after washing with PBS twice and thereafter lysed using a 4X Sample buffer (Invitrogen), 0.1M DTT (Sigma) and water. Supernatants were concentrated using Amicon Ultra 50 K centrifugal filters (Millipore UFC505024), and the cell lysate was sonicated. Both supernatants and lysate were boiled and resolved by 10% sodium dodecyl sulfate polyacrylamide gel electrophoresis (SDS-PAGE), and then electro transferred to a polyvindiline difluoride (PVDF) membrane (Millipore). The membrane was blocked in 5% skimmed milk and incubated with the HMGB1 antibody (rabbit, polyclonal, Abcam ab 18256); the membrane was then rinsed several times with TBST, incubated with a horseradish peroxidase (HRP)-conjugated secondary antibody (Abcam ab6721), rinsed again with TBST and finally developed using WB Luminol Reagent (Santa Cruz Biotechnology, Heidelberg, Germany).

### Release of cytochrome-C

A375 cells were seeded as in HMGB1 studies, and treated with 35 μM LTX-315 for different time points (5, 15, 45 min). Supernatants were collected and concentrated as with HMGB1 studies, and samples from the supernatants were analyzed using a 4.5-hour solid form Cytochrome C- Elisa kit (R&D Systems, USA, #DCTC0) following the manufacturer's instructions. Shortly thereafter, a 50% diluted sample was analyzed and the optical density was determined using a microplate reader set at 450 nm. This reading was then subtracted from the reading at 540 nm. A standard curve was generated for each set of samples assayed. Samples were run in four parallels, and the cytochrome-C released into the supernatant was expressed as a fold over the level of cytochrome-C in the supernatant of untreated cells.

### Release of ATP

The supernatant of LTX-315-treated A375 cells was analyzed using an Enliten ATP luciferase assay kit (Promega, USA). Cells were seeded as described for ROS assay, and treated with LTX-315 (35 μM) for 1–15 minutes; each experiment was performed with two parallels and conducted three times. Negative controls were untreated A375 cells exposed to serum-free medium alone. Samples diluted 1:10 were analyzed with a Luminoscan RT luminometer according to the manufacturer's protocol.

### Statistical analysis

All data represent at least two independent experiments with at least two parallels, which were expressed as the mean ± SD. Cytochrome-C were conducted twice with four parallels. ATP luciferase assay was conducted three times with two parallels. ROS assay were conducted twice with two parallels. Cytochrome-C release and ATP release data was compared using one-way ANOVA and a multiple comparison test (Tukeys Multiple Comparison Test), and we considered the *P*-value < 0.05 statistical significant.

## SUPPLEMENTARY VIDEO


