# Supplementary material for: The oncolytic peptide LTX-315 induces cell death and DAMP release by mitochondria distortion in human melanoma cells
Source: Oncotarget. 2015 Oct 13;6(33):34910–23. doi: 10.18632/oncotarget.5308 (PMC4741498; doi:10.18632/oncotarget.5308)
Supplement: Supplementary file 1 [file oncotarget-06-34910-s001.pdf]

SUPPLEMENTARY VIDEO

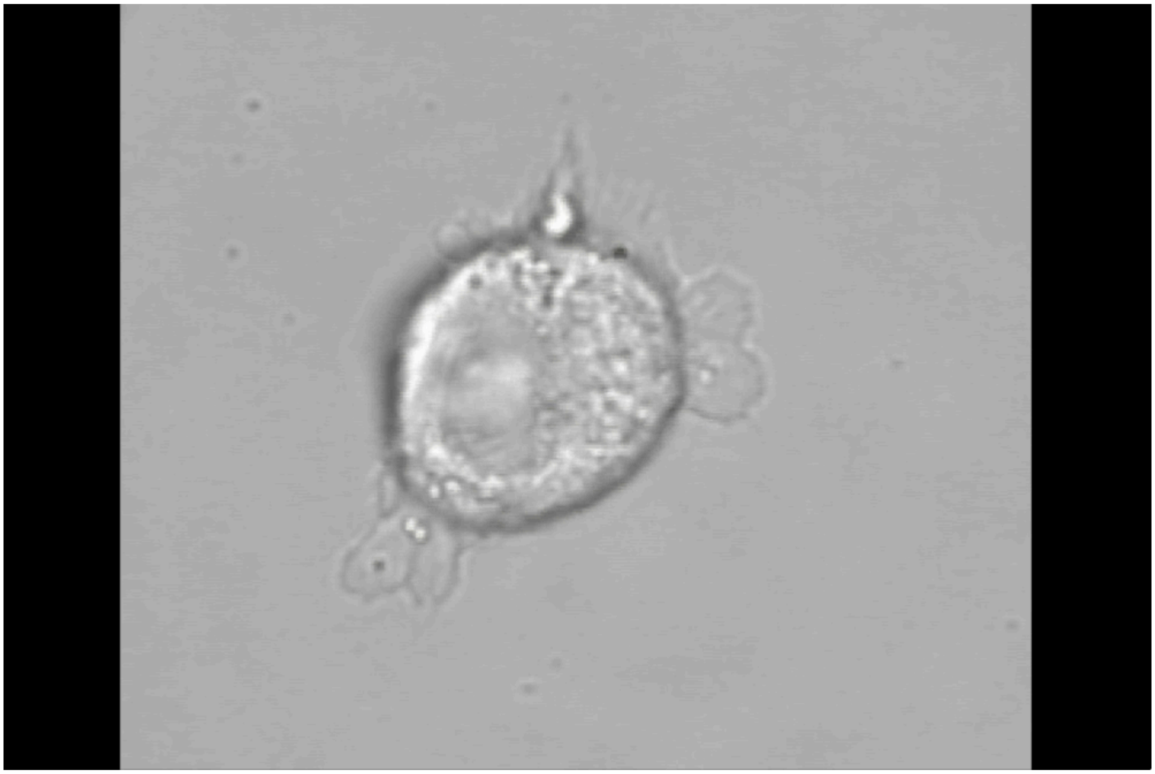

Supplementary Video: A375 cells were seeded at 10,000 cells/well in a complete medium in Nunc Lab-Tec 8-wells chambered covered glass (Sigma) pre-coated with 25µg/ml human fibronectin (Sigma) that were allowed to adhere overnight. Cells were washed twice with a serum-free RPMI, treated with peptide (17µM) dissolved in RPMI and investigated using Bright Field on a Leica TCS SP5 confocal microscope, with a 63X/1.2W objective. The microscope was equipped with an incubation chamber with CO_2 and temperature control.
